# Variation in management of humeral and clavicular shaft fractures amongst fellowship trained orthopedic traumatologists

**DOI:** 10.1186/s12891-020-03639-x

**Published:** 2020-09-18

**Authors:** Behnam Sharareh, Christopher Perkins

**Affiliations:** Department of Orthopedic Surgery, Baylor College of Medicine, 7200 Cambridge St. Suite 10A, Houston, TX 77030 USA

**Keywords:** Humerus, Clavicle, Operative management, Conservative management, Surgeon preference

## Abstract

**Background:**

There exists a wide variety of opinions on the appropriate management of diaphyseal humeral and clavicular fractures amongst orthopedic surgeons.

The purpose of this study is to determine if there is a preference amongst orthopedic traumatologists on treatment of diaphyseal humerus and clavicle fractures with respect to various patient populations.

**Methods:**

A 6-question survey was created using Surveymonkey.com and distributed via the Orthopedic Trauma Association (OTA) website to fellowship trained orthopedic surgery traumatologists to survey the preferred management of a simple oblique middle 1/3rd diaphyseal humerus fracture and a middle 1/3rd displaced diaphyseal clavicle fracture in the following 3 clinical settings: a healthy laborer, an older patient with co-morbidities, and if the surgeon themselves sustained the injury. The ratio of operative to non-operative management was calculated for all 6 questions. A chi-square value was performed to determine if the results are clinically significant based on the clinical scenario.

**Results:**

There was 56 responses to the survey that were included in the analysis. Overall, there was a statistically significant trend towards surgical management of the surgeon’s own diaphyseal humerus fractures (55%) compared to that of healthy patients (41%) and those with medical comorbidities (21%) (*p* = 0.02) A similar trend was noted for operative management for diaphyseal clavicle fractures by the surgeon on their own fractures (43%) compared to that of healthy patients (38%) and those with medical comorbidities (18%) (*p* = 0.02).

**Conclusion:**

While there are an increasing number of relative indications for treatment of diaphyseal humerus shaft and clavicle fractures, the results of this survey indicate that fellow-ship-trained orthopedic trauma surgeons prefer surgical management of simple humerus and clavicular fractures in young, healthy patients as well as in themselves.

## Background

Diaphyseal humeral fractures are one of the most common fractures sustained in the adult population, comprising of 2-5% of all fractures and roughly 20% of all fractures involving the humerus [1, 2]. Treatment of diaphyseal humerus fractures can be operative or non-operative. Decision making on treatment is multi-faceted and depends on location of injury, fracture morphology, accompanying neurovascular injuries, open or closed injuries and patient-associated factures. Non-operative treatment consists of coaptation splinting and functional bracing which was made popular by Sarmiento et al. in 2000 showing union rates as high as 94% for certain fractures treated in this manner [3]. Operative management involves plate and screw fixation, intramedullary nailing, and in rare cases, external fixation [4–6]. General consensus on absolute indications for operative fixation of diaphyseal humerus fractures include open fractures, concomitant brachial artery or plexus injury, and ipsilateral forearm fracture (“floating elbow”). There are also “relative” indications which include the polytrauma patient to allow for earlier weightbearing, bilateral humerus fractures and pathological fractures. While there is much agreement regarding treatment of humeral shaft fractures if the above criteria met, the indications for treatment with respect to fracture morphology are not universally agreed upon. Traditionally, fractures that were distracted at the fracture site, transverse patterns and those outside the post-reduction alignment criteria of: < 20 degrees anterior/posterior angulation, < 30 degrees varus/valgus angulation and < 3 cm shortening, were considered for operative management [2]. However, there still remains significant controversy on which fractures are indicated for operative management based purely on fracture morphology and displacement.

Similarly, clavicle fractures are very common in the adult population, comprising of 2-4% of all fractures [7]. Like diaphyseal humerus fractures, indications for treatment of diaphyseal clavicle fractures depends on associated injuries, soft tissue appearance and fracture morphology. The absolute indications for operative treatment of clavicle fractures include open fractures, floating shoulder (concomitant scapular neck fracture) with > 1 cm shortening of scapular neck, neurovascular injury and skin tenting or skin compromise. The relative indications for operative management include the polytrauma trauma, brachial plexus injury, those with significant shortening > 2 cm, and unstable fracture patterns with involvement of coracoclavicular ligaments. There, however, is a mixed consensus on management of diaphyseal clavicular fractures that are simply significantly displaced [8–10].The purpose of this study is to determine if there is a preference amongst orthopedic traumatologists on treatment of diaphyseal humerus and clavicle fractures that do not meet traditional criteria for operative management with respect to fracture displacement and morphology. This survey study was sent to orthopedic traumatologists to determine if there is bias between the preference of treatment of diaphyseal humerus and clavicle fractures and if these preferences change based on patient age, presence of comorbidities or if the injuries were sustained by the surgeons themselves.

## Methods

A 6-question survey was created (SurveyMonkey, San Mateo, California) and distributed via the Orthopedic Trauma Association (OTA) website to fellowship-trained orthopedic surgery traumatologists. The survey was left on the website for a total of 6 months. There are approximately 1500 fellowship trained orthopedic surgery traumatologists practicing in the US that are active members of the OTA. A copy of the survey is included below (Additional file 2). The survey was broken down to 3 pages with 2 questions per page involving 2 radiographs. The first question involved a radiograph of simple spiral midshaft humerus fracture with 20 degrees of angulation in the sagittal plane and 25 degrees in the coronal plane with 1 cm of shortening. The angle and shortening numbers are not provided in the stem. The authors are given a clinical scenario of a healthy, working individual who sustains the injury and if they would prefer to proceed with initial operative or non-operative management. There was no mention of a closed reduction attempt in this scenario. The 2nd question involves a radiograph of a left diaphyseal clavicle fracture with > 100% of displacement but no shortening. The clinical scenario given is the same as in Question 1. After answering the first 2 questions and pressing “next,” the responders were unable to go back and change their answers to the previous questions. The responders also had to answer both questions on the page in order to be able to advance to the following page. Question 3 and 4 involve the same radiographs as in questions 1 and 2 but with the clinical scenario of an older patient with medical comorbidities. Finally questions 5 and 6, which also involved the same 2 radiographs as the prior 4 questions, gives the clinical scenario of the surgeon (the survey taker) themselves sustaining the injury. The ratio of operative to non-operative management was then calculated for all 6 questions. A chi-square value was then performed to determine if the results are clinically significant based on the 3 clinical scenarios with humeral fractures and then independently, the 3 clinical scenarios with clavicular fractures (see additional file “Data”).

## Results

The survey was placed on the OTA website for 6 months. The number of responses to the surveys was 58. Of the 58 responses, 56 responders completed the survey in its entirety (97%). The 56 complete responses were included in the analysis (Table 1). For diaphyseal humerus fractures, there was a statistically significant (Chi value 5.02, *p* = 0.02) difference between those who preferred operative management for a “healthy, working” patient (41%) compared to those who preferred operative management for an older patient with medical comorbidities (21%). There was also a statistically significant (Chi value 3.2, *p* = 0.049) difference between those who preferred operative management for a “healthy, working” patient (41%) compared to those who preferred operative management for themselves (55%). Similarly, for diaphyseal clavicle fractures, there was a statistically significant (Chi value 5.40, *p* = 0.02) difference between those who preferred operative management for a “healthy, working” patient (38%) compared to those who preferred operative management for an older patient with medical comorbidities (18%). There was no statistically significant (Chi value 0.3, *p* = 0.56) difference between those who preferred operative management for a “healthy, working” patient (38%%) compared to those who preferred operative management for themselves (43%). Finally, there was no statistically significant difference between the percent who preferred operative treatment for diaphyseal humerus and clavicle fractures within each clinical scenario.

**Table 1:** Responses of the 6-question survey sent to OTA orthopedic surgery traumatologists (*n* = 56 complete responses)

Preferred treatment	Question 1	Question 2	Question 3	Question 4	Question 5	Question 6
Operative	23	21	12	10	31	24
Non-operative	33	35	44	46	25	32
% Operative:	41%	38%	21%	18%	55%	43%

## Discussion

There exists a wide variety of differing opinions with respect to management of diaphyseal humerus and clavicle fractures. While management of diaphyseal humerus fractures initially focused on non-operative management, more recent data has slightly shifted to surgical management of these fractures with importance of identifying various fracture morphologies that risks failure of non-operative management [11, 12]. In our current study there was a trend of orthopedic trauma surgeons who stated they would prefer surgical intervention on their own simple pattern diaphyseal humerus fractures (55%) and in healthy patients (41%) compared to those with medical comorbidities (21%).

The data on treatment of diaphyseal humerus fractures has continued to evolve over the last 20 years with increased prevalence of studies favoring operative management [11–14]. Denard et al. performed a retrospective review of 213 patients with humeral shaft fractures who underwent treatment with a functional brace versus ORIF to determine the difference between non-union, malunion, incidence of radial nerve palsy and elbow range of motion [11]. The authors noted a statistically significant decreased rate of non-union and malunion in the operative group compared to the non-operative group and no difference between incidence of infections, radial nerve palsy, time to union or elbow range of motion. This study, however, did not cite fracture morphology, and patients with polytrauma were more likely to undergo surgical treatment. Hosseini Khameneh et al. performed a randomized clinical trial of 60 patients who were randomized to ORIF versus Sarmiento brace and noted a mean union time of 13.9 weeks in the operative group versus 18.7 weeks in nonoperative group, which was statistically significant [12]. They also noted no difference between DASH scores and no difference between risk of non-union between the groups. Ma et al. in a retrospective chart review noted the decreased healing time in operative management (10.4 weeks) compared to nonoperative management (15.7 weeks) and noted that 93% of patients in the plate fixation group were satisfied with the appearance of their arm compared to the 77% in the non-operative group [13]. Furthermore, Ali et al. did a retrospective review of 207 humerus shaft fractures over a 5 year period that were treated non-operatively to determine if there was a particular fracture type that went on to non-union (which was defined that absence of bony union at 1 year or delayed surgical fixation after 6 weeks) [14]. They noted an overall union rate of 83% with non-operative management but noted that proximal 1/3rd shaft fractures had a union rate of 76% compared to those of middle-third (88%) and distal third (85%). They noted that fracture comminution (3 or more parts) proceeded to union at a rate of 89% regardless of position. As such, the authors advocated for surgical management of proximal 1/3rd shaft fractures. The morphology of the diaphyseal fracture in our study was chosen to be one that was relatively simple (mid-shaft, non-comminuted, oblique) so the surveyors can simply just pick operative versus non-operative without considering controversial fracture morphology (transverse, distracted, proximal or distal 1/3rd). Surgeons still believed operative management was the preferred method for management of their own diaphyseal fracture in this setting.

With respect to polytrauma, the general consideration is to proceed with surgical management of humeral shaft fractures to allow for early weightbearing. Dielwart et al. performed a retrospective cohort study for patients with a Injury severity score (ISS) of > 17 randomized to those treated operative or non-operative by surgeon preference. The authors found 71 patients that met the criteria and noted no difference between union rates, time to union, or complication rates with respect to operative or non-operative rate [15]. The authors did note a statistically significantly earlier rate of weight bearing (9 weeks to 13 weeks) in favor of the operative group. The authors concluded that the polytrauma patient should be offered surgery for their humerus fracture based on demographics, ambulatory status, working status and expectations [15]. We did not assess polytrauma in our clinical scenarios for the decision to proceed with operative or non-operative management to avoid this as a potential bias.

Regarding diaphyseal clavicle fractures, there has been conflicting literature on rates of union as well as patient-oriented outcomes and earlier return to function with operative fixation compared to non-operative measures [16–21]. Liu et al. performed a meta-analysis on operative versus non-operative clavicle fractures and noted a statistically significant decreased relative risk of non-union (0.12), malunion (0.11) and neurological complication (0.45) with operative compared to non-operative management [17]. The authors did not differentiate data on fracture morphology or patient specific factors. Malik et al. also performed a systematic review to determine the effects of diaphyseal fracture shortening on shoulder function and nonunion rates in non-operatively managed fractures. They noted 4 randomized controlled trials, of which 3 reported no association between fracture shortening or shoulder outcome scores [18]. The authors concluded that there is no association between fracture shortening and union rates or shoulder outcome scores. In our survey, we chose a simple fracture pattern that showed 100% displacement without any shortening to avoid any potential bias towards shortening. The most recent randomized clinical trial comparing management of diaphyseal clavicle shaft fractures showed statistically significant increased rates of union at 9 months for the operative group compared to the non-operative group but no difference at 3 months [19]. The patient-reported outcomes and satisfaction rates were higher with operative intervention at 6 weeks and 3 months but no different to non-operative management at 9 months [19]. Contradictory to this, however, there has been 2 recent large Cochrane reviews that have stated there is insufficient evidence from randomized controlled trials to determine the most appropriate treatment of diaphyseal clavicle fractures and that individuals should be treated on a case to case bases [20, 21]. These Cochrane reviews did not consider the recent study by Ahrens et al. For our study, we noted a trend for operative management for diaphyseal clavicle fractures by the surgeon on their own personal fractures (43%) compared to that of healthy patients (38%) and those with medical comorbidities (18%).

There are several weaknesses and limitations to the study. Since the survey was placed on the OTA website for 6 months, and not specifically sent to the OTA orthopedic surgery faculty, an overall low number of responses was obtained and it is not entirely clear how many surgeons viewed the survey before deciding to respond. Furthermore, it is unclear if the surgeons that decided to respond to the survey had inherent bias towards management of such injuries operatively or conservatively. The average response rate of OTA survey studies according to the society is 5% and our response rate was slightly below this at 3.7% (56/1500). While our response rate is certainly low, it is not much lower than the expected response rate for this particular organization. Additionally, no surgeon-specific characteristics such as surgeon experience, surgeon age, surgeon practice model (academic, private practice), patient population treated, were obtained to determine if there are confounding variables that led to survey takers choosing particular answers. It should be noted that while OTA does not collect demographic information of their members, a recent survey found that the average age of orthopedic surgeons in the US is approximately 56 (https://www.aaos.org/aaosnow/2019/sep/youraaos/youraaos01/). Future survey studies would benefit from having surgeon demographic information embedded at the beginning of the survey.

## Conclusions

Overall, there appears to be a recent trend towards operative management of both diaphyseal humerus and clavicle fractures even in subgroups where traditional non-operative management has been the mainstay of treatment. In this survey study of orthopedic traumatologists, it appears surgeons would offer surgery to healthy patients and would prefer surgery themselves for these injuries. While there are an increasing number of relative indications for treatment of diaphyseal humerus shaft and clavicle fractures, the results of this survey indicate that fellow-ship-trained orthopedic trauma surgeons prefer surgical management of simple humerus and clavicular fractures in young, healthy patients as well as in themselves.

## Supplementary information

**Additional file 1.** Data: All survey responses are cited and the Chi-square results between each clinical scenario are provided.

**Additional file 2.** Copy of the survey questionnaire.

## Data Availability

Not applicable
