# Supplementary figures and images for: Variation in management of humeral and clavicular shaft fractures amongst fellowship trained orthopedic traumatologists
Source: BMC Musculoskelet Disord. 2020 Sep 18;21:618. doi: 10.1186/s12891-020-03639-x (PMC7499960; doi:10.1186/s12891-020-03639-x)

**Appendix A**

**
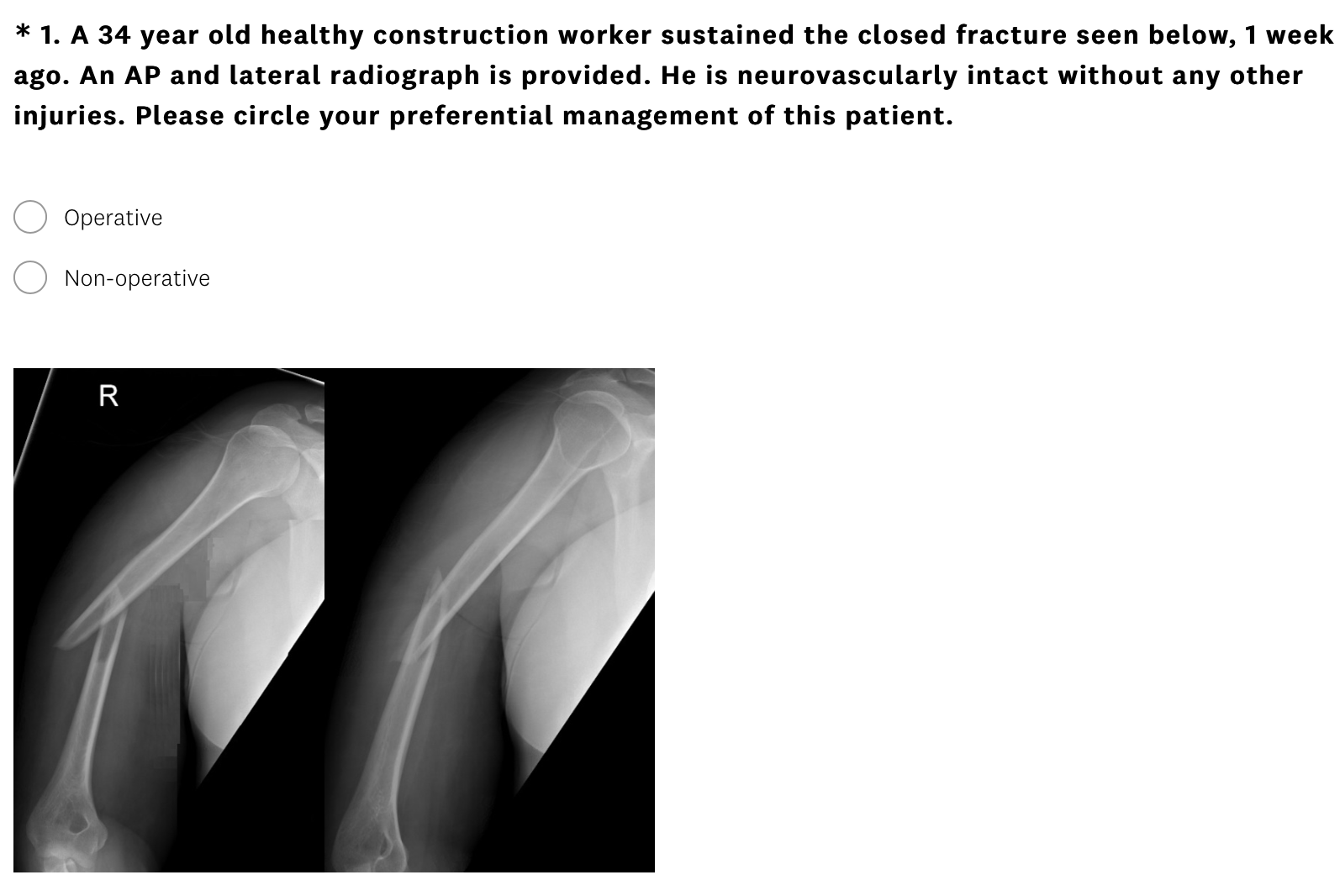
**

**
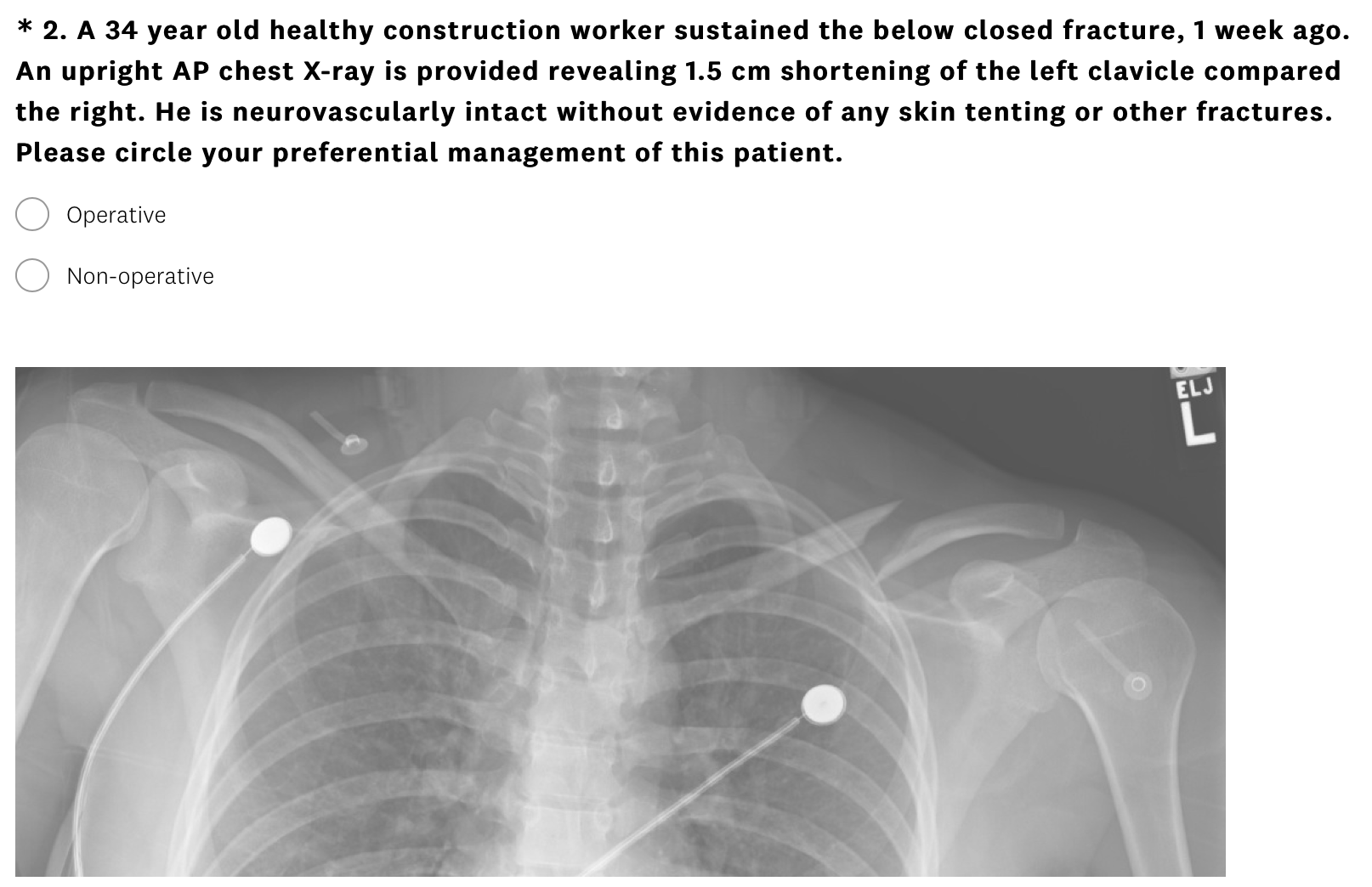
**

**
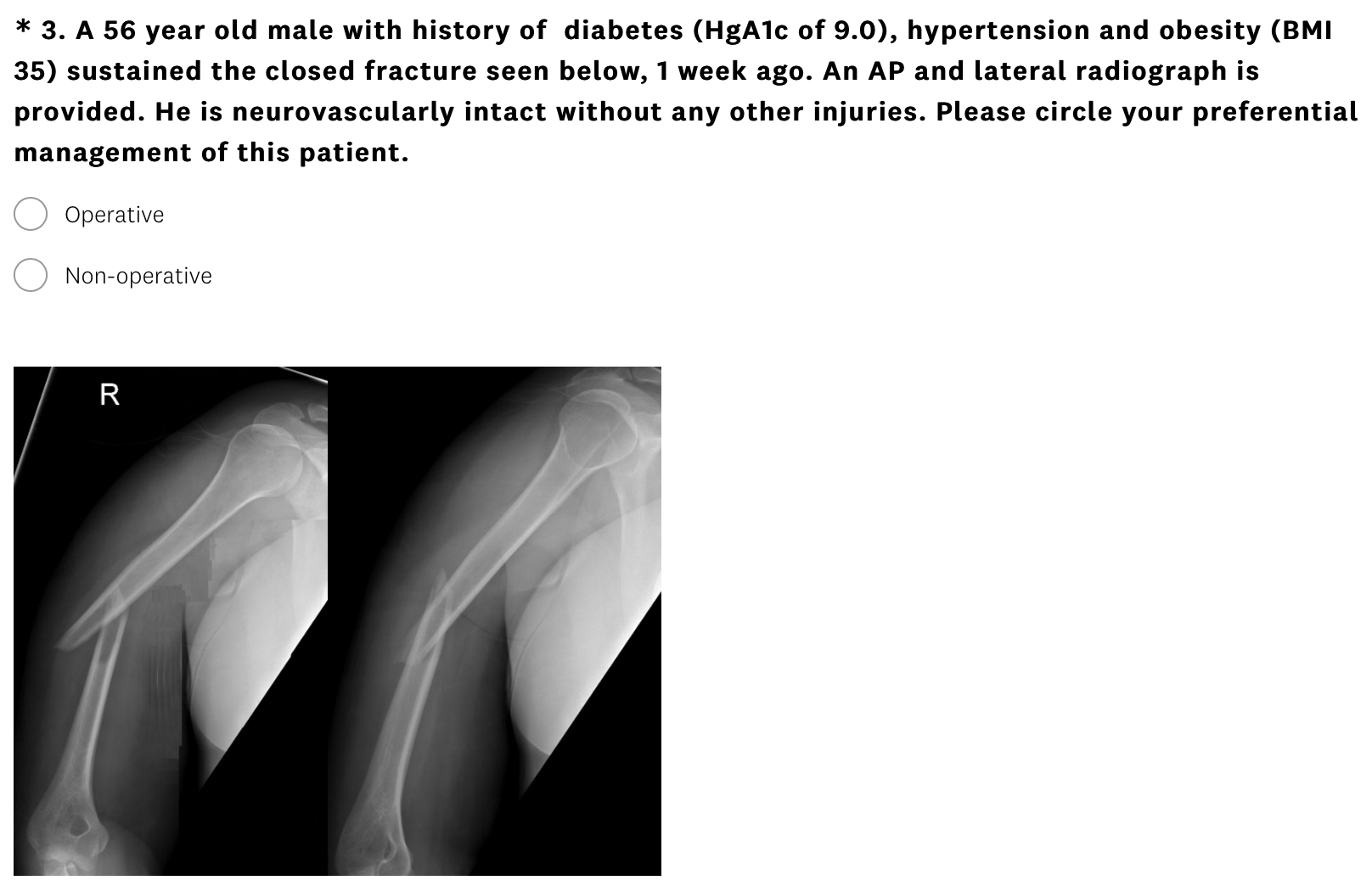
**

**
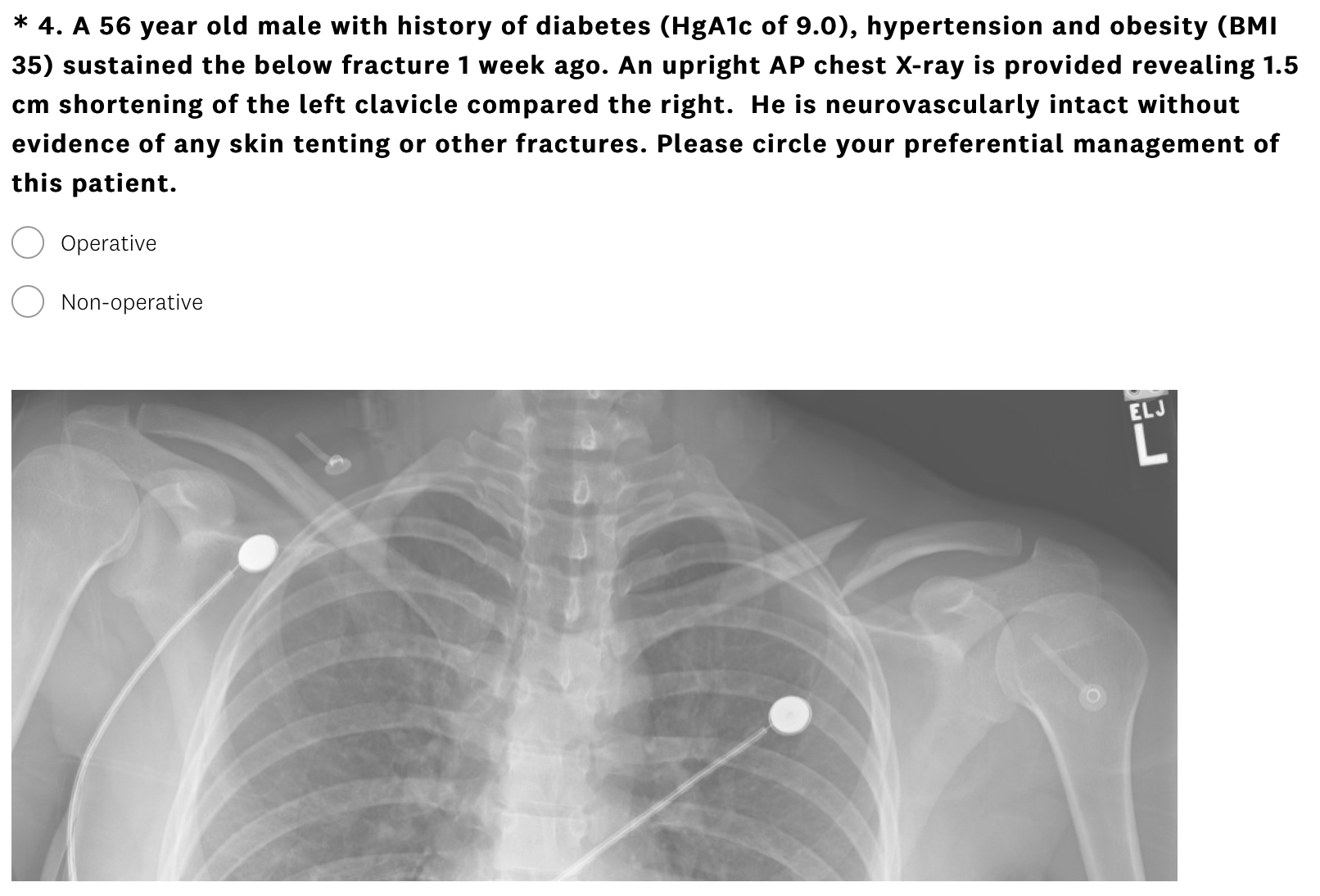
**

**
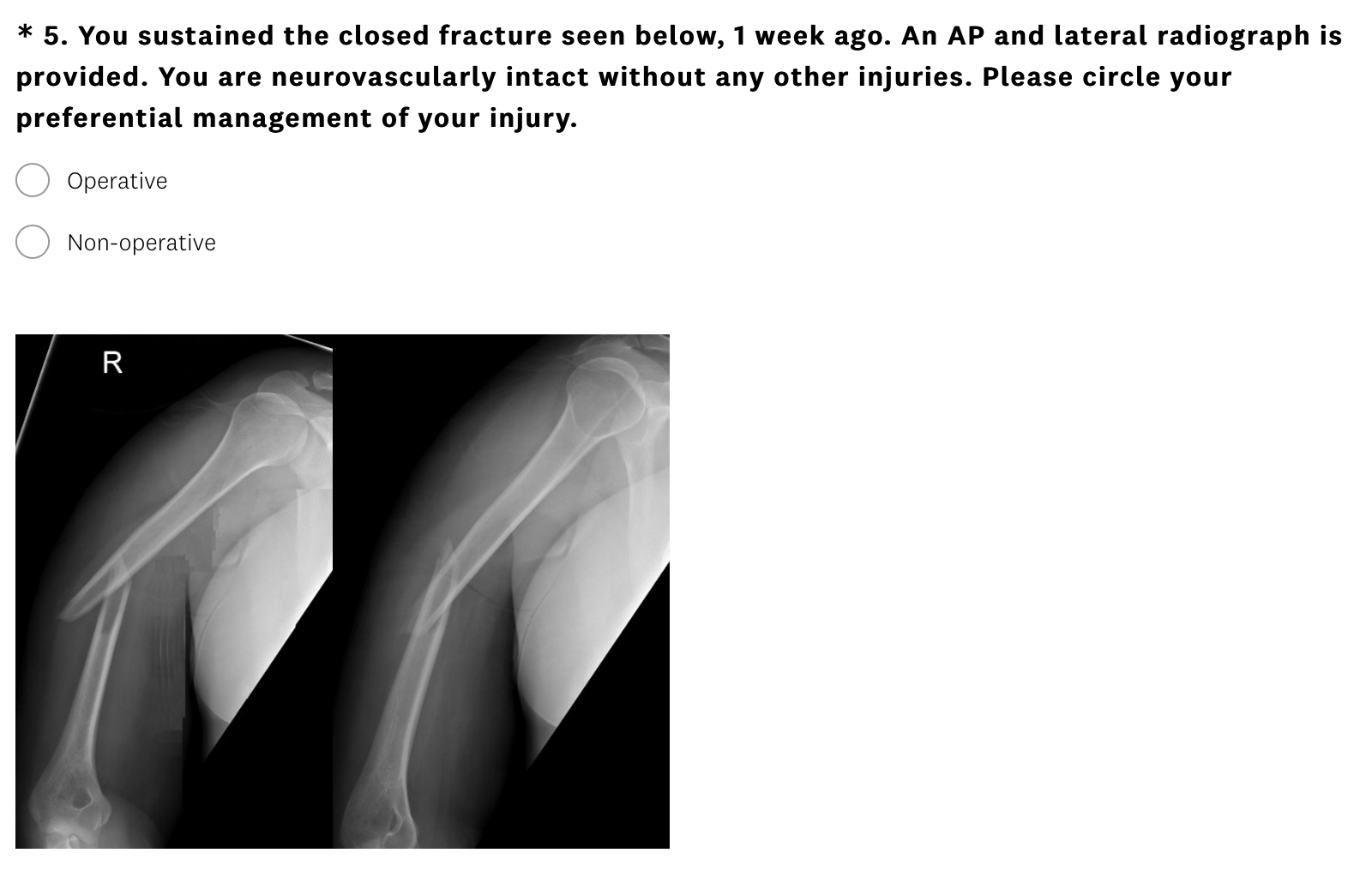
**

**
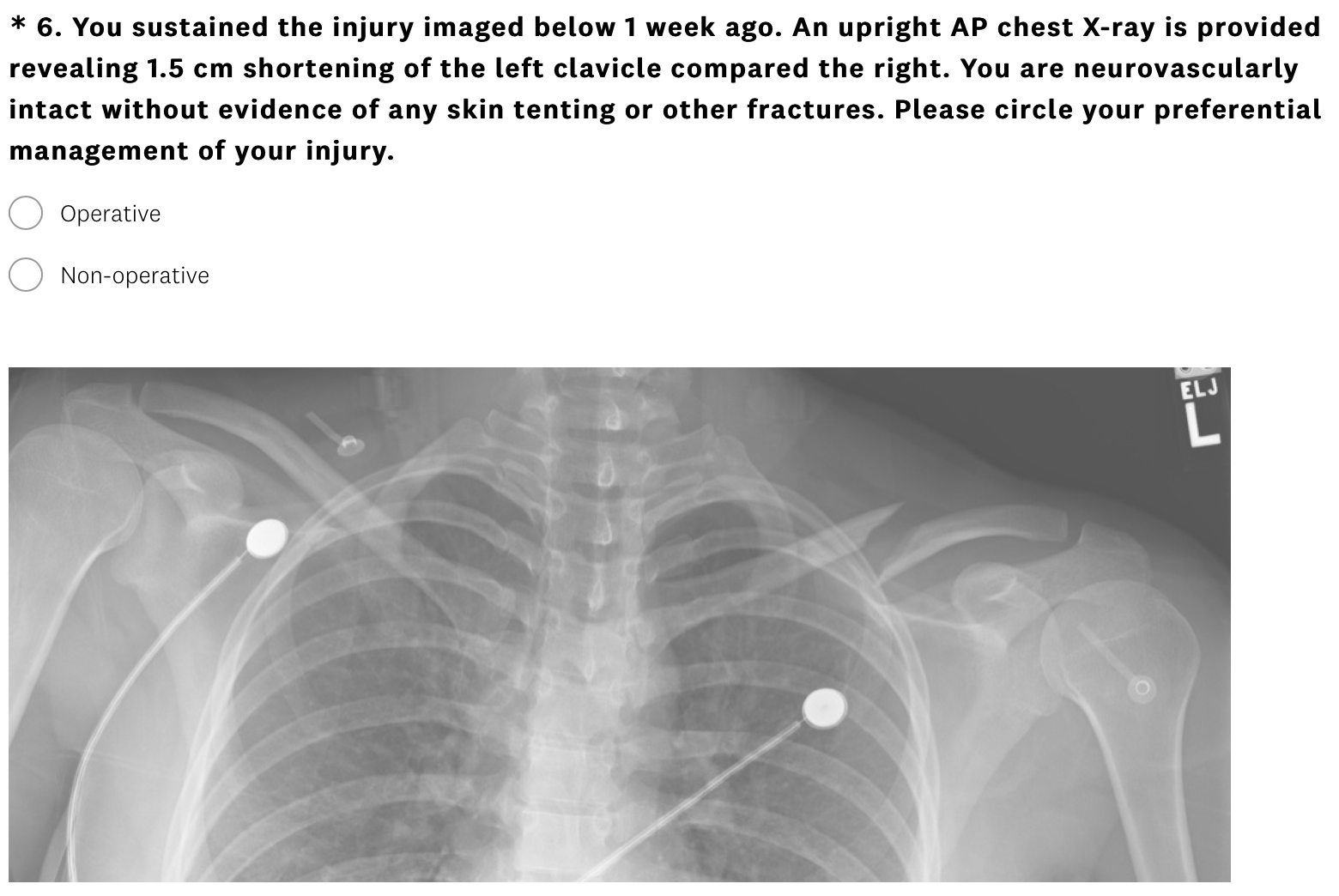
**

Supplement: Supplementary file 2 — Additional file 2. Copy of the survey questionnaire. [file 12891_2020_3639_MOESM2_ESM.docx]
